# Refractory Homicidal Ideation in a Young Adult Male With High-Functioning Autism Spectrum Disorder and Schizophrenia

**DOI:** 10.7759/cureus.65436

**Published:** 2024-07-26

**Authors:** Natasha R Patel, Numa Rehmani, Rachel Utter, Barbara Gracious

**Affiliations:** 1 Department of Psychiatry, Orange Park Medical Center, Orange Park, USA; 2 Department of Medicine, Edward Via College of Osteopathic Medicine-Carolinas Campus, Spartanburg, USA; 3 Department of Psychiatry, Hospital Corporation of America (HCA) Florida Orange Park Hospital, Orange Park, USA

**Keywords:** schizophrenia, cluster b personality disorder, erotomania, treatment-resistant psychosis, electroconvulsive therapy (ect), autism spectrum disorder and emotion, antisocial personality disorder, homicidal

## Abstract

Persistent homicidal ideation (HI), while not common among psychiatric disorders, can occur within multiple diagnoses in the Diagnostic and Statistical Manual of Mental Disorders. There is a growing global and national concern for homicide and homicide-related deaths. In this case report, we discuss refractory homicidal ideation in an 18-year-old male with the diagnoses of autism spectrum disorder (ASD) and schizophrenia and a history of Tourette’s syndrome and highlight the interplay of comorbidities and challenges in effective management and treatment.

## Introduction

Homicidal ideation (HI) refers to the thought about committing homicide, which can range from vague ideas to detailed plans without necessarily attempting or acting on them [1]. HI has significant implications for individual and public safety. There is growing global and local major public concern for homicide, both of which are deserving of attention. The global murder rate in 2022 was 6.1 per 100,000 people [2]. Homicide is particularly alarming within the adolescent population. The CDC reported in 2022 a total of 6,262 homicide-related deaths of adolescents aged 15-24, making homicide the third leading cause of death in that population [3]. Between 2015 and 2019, there was a 49% increase in juvenile court cases involving criminal homicide in the United States [4].

Homicidal ideation is more prevalent among individuals with psychiatric conditions [5]. In an analysis of the Nationwide Emergency Department Sample distributed by the Agency for Healthcare Research and Quality (AHRQ), homicidal ideation prevalence was found to be 0.25%, and multiple conditions were found to increase the likelihood of HI including but not limited to antisocial personality disorder (ASPD) (2,406%), schizoaffective disorder (1,821%), schizophrenia (1,143%), obsessive-compulsive personality disorder (921%), and other psychotic disorder (504%) [6].

Multiple studies have demonstrated a strong link between homicidal ideation in individuals with psychiatric conditions such as psychotic disorders, mood disorders, and personality disorders [7]. In a large study of homicide offenders, it was found that one-fifth suffered from a psychotic illness, half had a substance use or personality disorder, and over 90% had a psychiatric diagnosis [8].

Schizophrenia increases the odds of committing homicides by tenfold, as found in a Finnish study of more than 1,400 murders [9]. In a systematic review of 20 studies reporting on risks of interpersonal violence in individuals with schizophrenia, 9.9% were violent [10]. Fazel and Grann [8] also found that patients with severe depressive symptoms or mixed affective states were at a higher risk of having HI as well.

Multiple studies have cited associations between personality disorders and homicidal ideation and have characterized indicators for violence within these disorders, including impulsivity, anger, and paranoia [11,5]. Carbone et al. [6] found a powerful association between personality disorders and homicidal ideation, and the largest effect was for antisocial personality disorder, which conferred a 2,406% increased likelihood of homicidal ideation. Black et al. [11] discuss that the pervasive pattern of deceitfulness, impulsivity, and irritability in individuals with ASPD increases the likelihood of violent thoughts and behaviors.

Violence is also seen in patients with autism spectrum disorder (ASD), as noted in a study by Kanne and Mazurek [12], which found that 30% of children and adolescents with ASD exhibited aggressive behaviors. Violent behaviors are not inherent features of ASD, but there is growing recognition that some individuals with ASD experience homicidal ideation or violent thoughts and behaviors. Individuals with ASD who have a history of trauma are also at greater risk for developing violent thoughts or behaviors [13].

## Case presentation

History of present illness

The patient is an 18-year-old Caucasian male with a documented history of autism spectrum disorder (ASD), schizophrenia, and Tourette’s syndrome, who was brought to the hospital by his family for homicidal ideation in February 2024. The patient presented to the ED accompanied by his family for paranoia and HI, expressing a plan to run away and kill people and animals. The patient’s family reported that the patient had prepared a bag containing a kitchen knife, intending to carry out these actions. This prompted the family to bring the patient in for evaluation. Initially, he denied any auditory or visual hallucinations. However, on subsequent evaluation in the inpatient unit, he reported hearing voices, sometimes his own and other times unfamiliar ones, telling him to hurt people. During the initial inpatient assessment, the patient disclosed a history of killing small reptiles, such as frogs, but denied harming any people. He also shared fantasies about hurting other people, including contemplating violence while staring at their jugular veins. He reported intrusive thoughts and hallucinations, struggling to distinguish one from another. He reported visual hallucinations of black dots, stating that it was similar to an aura. He also reported olfactory hallucinations of coconut and peanut butter but denied any associated thoughts or references to these smells. The patient also gave a recent history of self-injurious behavior of cutting his arm with a knife and then putting hand sanitizer on the wound one month prior to admission. He stated that he did this because he enjoyed the sight of his blood and the burning sensation. He denied nausea, vomiting, headache, chest pain, shortness of breath, constipation, diarrhea, or abdominal pain. Screenings for major depressive disorder (MDD), mania, and post-traumatic stress disorder (PTSD) were all negative.

Further discussion with the patient’s mother revealed a potential pattern of symptom exacerbation approximately three weeks after receiving aripiprazole as a long-acting injectable (LAI), suggesting a need for a closer monitoring of medication efficacy and potential heightened metabolism lowering his response to treatment. The patient had been taking aripiprazole LAI for a few months prior to admission and was recently prescribed oral aripiprazole by his outpatient psychiatrist as an adjunct due to the subtherapeutic benefit from LAI.

Past psychiatric history

The patient had a documented psychiatric history of schizophrenia, Tourette’s syndrome, and autism spectrum disorder and previous involuntary hospitalizations. At least two admissions were within six months of the most recent admission for homicidal and suicidal ideation. He had a history of suicide attempts, with his most recent attempt being an overdose on oral olanzapine, one month prior to his current admission. He also reported a history of violence toward animals, specifically killing frogs and lizards with a knife. The patient had no history of substance abuse. He was under the care of an outpatient psychiatrist regularly, and his oral psychiatric medication regimen on admission included oral sertraline 50 mg daily, oral aripiprazole 20 mg at bedtime, and oral oxcarbazepine 300 mg twice daily (BID). He was also receiving aripiprazole 400 mg LAI every 28 days.

Past medical history

The patient’s past medical history included hypothyroidism, hypertension (HTN), reported history of pituitary adenoma via magnetic resonance imaging (MRI), and membranoproliferative glomerulonephritis (MPGN) type III. His current medications also included levothyroxine 50 mcg in the morning for hypothyroidism and lisinopril 5 mg daily for hypertension.

Family history

The patient’s mother had been diagnosed in the distant past with major depressive disorder (MDD) and had a history of a suicide attempt. His sister had MDD as well. His father had PTSD and depression and was a disabled veteran. His brother had been diagnosed with ASD, schizophreniform disorder, and seizure disorder. His maternal grandmother and maternal uncle had obsessive-compulsive disorder (OCD).

Social history

The patient resided with his mother, stepfather, younger brother, and younger sister and also had an older brother. He had a mostly good relationship with his mother, stepfather, and younger brother. He mentioned that his stepfather occasionally showed anger by yelling but denied episodes in recent years. His biological father was a veteran with disabilities and severe PTSD who had left the family 12 years prior. The patient was enrolled in and had been attending college full time primarily online while living at home. His hobbies included writing fiction stories, of which many included violence. He denied being in a romantic relationship, had no children, and did not work. He reported his mother and brother as his main sources of emotional support. He described a generally happy childhood, except for a brief period when his mother was hospitalized for a suicide attempt. During that time, he lived with his paternal grandparents; he mentioned that there was an episode in which his grandparents had him and his sister sleep with no clothes on in the bed with them. He denied being inappropriately touched by them. The patient denied any access to firearms but did admit to access to knives.

Complete hospital course

The initial treatment plan upon admission included the initiation of his home oral medications sertraline 50 mg daily and oxcarbazepine 300 BID, as well as lisinopril and levothyroxine. He also started taking aripiprazole 10 mg orally a few days prior to this admission per recommendation from his outpatient psychiatrist due to his increasing homicidal ideation. The frequency of aripiprazole LAI 400 mg was changed to every three weeks instead of four weeks due to concern for worsening symptoms and insufficient therapeutic response three weeks after the last dose.

Despite this change in earlier LAI dosing, the patient’s symptoms of paranoia and homicidal ideation persisted, leading to an increase of the oral aripiprazole to 15 mg at bedtime. These symptoms included a reported plan to kill a larger animal such as a possum, a household pet, or a person upon being released from the unit. He denied an intent to hurt others in the hospital. He described an intent to conceal the act and evade legal consequences by hiding the body. On discussing how he could evade police, he indicated that he would have to “take them out as well, which would be very difficult.” Given these symptoms and the reported history from the patient of violent thoughts and the killing of small animals, we did consider a diagnosis of antisocial personality disorder, although, during his hospital stay, he did not receive this diagnosis. He had not previously engaged in antisocial behavior other than the killing of small reptiles and amphibians.

The patient was then started on oral loxapine 25 mg at bedtime in addition to aripiprazole, as an augmentation strategy for irritability and aggression in ASD [14]. Additionally, loxapine is similar to clozapine without the need for weekly laboratory work and with fewer potential side effects. The loxapine dose was increased to 25 mg twice a day after seeing an initial signal of response in his psychotic symptoms but continued residual symptoms of paranoia and homicidal ideation. Since oxcarbazepine is a cytochrome P450 (CYP) 3A4 inducer, it was discontinued due to possible inductive effects on the metabolism of aripiprazole, which is metabolized by the CYP3A4 pathway [15]. Lithium was also considered, but due to his concurrent condition of MPGN type III, the nephrotoxic adverse effects of lithium were of concern, and it was ultimately excluded from the treatment plan at that time. Residual symptoms also involved clearer intentions and plans, including a plan of hurting his paternal grandmother and another plan to kill a random person and hide the body. The patient also reported feeling nervous about therapy dogs on the unit coming in and had urges to harm them.

On day 9, the patient experienced transient symptoms of elevated mood, with disorganized thinking, increased paranoia, and visual hallucinations. These symptoms improved following the administration of his bedtime dose of oral aripiprazole but recurred in the morning, suggesting a more fluctuating pattern. Sertraline was discontinued due to this report of mood change. He was given his monthly dose of aripiprazole LAI, which resolved his homicidal ideation, paranoia, and hallucinations for two days. A trial of lithium was then started to target HI and paranoia after consultation with the patient’s nephrologist; however, again, he had no response. He then reported HI toward his family with thoughts of starting a house fire with his family inside the home. During an interview on hospital day 13, the patient revealed symptoms of erotomania with thoughts that a young staff member would return his love if they knew him better. He admitted to searching for the staff member’s address online and attempted to get a rideshare to their address prior to the current admission. This was confirmed by his mother, who searched his phone and found evidence of the rideshare call. Later, the patient’s mother also found other notes on his phone that indicated increasing preoccupation with this staff member after discharge from a prior hospitalization. On day 14, the patient made suicidal gestures by hiding and then holding a broken plastic knife to himself, attempting to bargain leaving the hospital. The patient’s mother expressed considerable concern for her family and stated that she could not take him home with worsened ideation to harm himself and others, including specific thoughts and plans to harm family members and burn down the house.

The lack of signal of response to various oral and injectable medications and the fluctuation of symptoms in both intensity and frequency without sustained remittance prompted a discussion with the treatment team, mother, and patient of alternative treatment options. A decision was made to transfer the patient to a different facility due to the availability of electroconvulsive therapy (ECT). Another reason was the availability of a monitored private room at the transfer facility as a safer option for the patient, other patients, and staff.

Following the transfer, the patient was started on clozapine due to persistent erotomania and HI. The patient was switched from lithium to lamotrigine after the consultation and recommendation of a nephrologist at the new facility. Aripiprazole was switched to olanzapine due to continued auditory hallucination and the report of agitation. This switch ultimately did improve both hallucinations and agitation. A neurology consultation was obtained, which noted a history of one isolated seizure in childhood without a history of any long-term anti-seizure medication or recommendations for initiation. He was given clearance for ECT without restriction from the neurology team.

The patient received 12 sessions of bilateral ECT scheduled three times a week to date of this report. The ECT quality was described as high amplitude and good progression with modest stimulus. The seizure and treatment quality was reported as excellent; however, the patient’s symptoms did not completely improve. The patient denied delusions, auditory and visual hallucinations, and homicidal ideations; however, the staff and psychiatrists observed the patient to be responding to internal stimuli. To date of this report, the patient continued to receive ECT treatments while inpatient, and there had not been a petition started for state hospitalization.

## Discussion

This patient’s case emphasizes the complexity and challenges associated with managing refractory homicidal ideation with ASD and other comorbidities. The patient’s symptoms persisted despite several psychopharmacology trials and eventually required ECT, an intensive intervention. Therapeutic strategies such as cognitive behavioral therapy (CBT) were attempted; however, it was evident that the patient had some relief of distress when he had homicidal ideation periods, which were unpredictable and related to paranoid thoughts, suggesting a possible self-soothing-type effect. Cognitive behavioral therapy (CBT) in an intensive setting such as residential treatment is a future option.

The patient’s history of violence toward animals and his observed refractory homicidal ideation even with the improvement in his psychosis may point to a potential comorbidity of antisocial personality disorder (ASPD). The criteria for ASPD include a continuous pattern of disregard and violation of the rights of others, since 15 years old, with three of the following: failure to conform to laws and social norms, deceitfulness, impulsiveness, irritability and aggressiveness, reckless disregard for the safety of oneself and others, consistent irresponsibility, and the lack of remorse. The individual must be at least 18 and show signs of conduct disorder before the age of 15 [16]. We understand from Carbone et al. [6], as noted above, that there is a strong link between ASPD and homicidal ideation. The patient’s mother denied an earlier childhood history of symptoms of conduct disorder; however, the patient admitted to the onset of thoughts and urges to harm others along with hallucinations beginning at the start of middle school, possibly when pubertal hormone changes begin occurring. The patient indicated a chronic pattern of a lack of remorse. Despite his mother’s history, there appears to have been strong evidence for ASPD traits in the patient’s report. Of special note, the mother was unable to give any information about the patient’s biological father and his family, stating that it had been many years since she had any contact with him.

Additionally, the patient has a comorbidity of MPGN type III and a history of seizure in childhood. It is unknown if the patient’s MPGN type III played any role in his psychopathy. There are studies on diseases that involve the kidney and also affect the brain such as hemolytic uremic syndrome (HUS), thrombotic thrombocytopenic purpura (TTP), and postinfectious glomerulonephritis (PIGN). HUS and TTP create ischemic manifestations in the kidneys and brain [17], and neurological and cerebral symptoms are frequently seen in PIGN [18]. However, sufficient research does not exist on whether there is any link between the disease process of MPGN type III and the development of psychopathy. Inflammatory markers and basic workup including urine drug screen, urinalysis, blood alcohol level, comprehensive metabolic panel (CMP), CBC, acetaminophen level, syphilis enzyme immunoassay, lipid panel, hemoglobin A1c panel, thyroid-stimulating hormone (TSH) panel, celiac disease panel, and zinc, iron, and magnesium panel for medical causes of early-onset psychosis were negative. However, there was an increase in ESR related to his MPGN during a trial off his lisinopril, which had kept his disease in good control per the consulting nephrologist. The patient did receive a neurology workup due to one isolated seizure in childhood; however, he was cleared for ECT and subsequently tolerated ECT without complications.

As discussed above, traumatic experiences are also a risk factor for violence in people with ASD. The patient recalled multiple traumatic experiences in his childhood, including his mother’s suicide attempt, a verbally abusive stepfather, and an episode where his grandparents had him sleep naked with them. Although the patient experienced emotional responses to the trauma, there was no clear evidence of PTSD. Traumatic experiences can be difficult to process, especially in patients with ASD who have a theorized lower threshold to cope with stressors [19]. These individuals struggle to understand context or interpret social cues. As a result, they may overly fixate on nonessential details, misattribute the source of their negative feelings, and lack the flexibility to consider alternatives to violence as a coping mechanism. These factors increase the risk that these individuals may engage in violence [13]. Beyond ASPD, narcissism related to childhood parental grievances may also contribute to feelings of rage and a lack of empathy for others. What role his infatuation with a staff worker played is unclear, but we could not rule out the possibility of his seeking admission to be closer to that person, until after the transfer, where his homicidal thoughts persisted.

It is important to note the challenges in treating psychiatric disorders in an individual with comorbid ASD, MPGN with HTN, and psychosis. When choosing a medication, particularly psychotropics, the aim is to select the proper treatment regimen with careful consideration of possible interactions and toxicities. Due to the patient’s preexisting diagnosis of MPGN type III, which primarily affects the kidneys, a consideration of lithium’s nephrotoxic effects was made. Ultimately, the patient’s severe and refractory homicidal ideations called for alternative psychopharmacological agents, and a nephrology consult ultimately agreed that the potential benefits of a lithium trial could outweigh the risks. Due to the patient’s lack of continued response despite multiple pharmacological treatments, including a short trial of lithium, without meaningful remittance from symptoms, ECT was considered as a treatment given his baseline condition of psychosis.

Although the patient had a psychiatric comorbidity of schizophrenia, the episode of psychosis was distinguishable from his refractory HI. Despite showing signs of improvement in psychotic symptoms, the patient continued to experience HI. This was most evident when his thought processes became clearer, which unfortunately came with clearer and more detailed homicidal planning. In regard to other psychiatric comorbidity, OCD was also considered as a cause for his refractory homicidal ideation, but ultimately, the patient neither had significant distress due to intrusive thoughts nor did make any attempt to suppress or neutralize the intrusive thoughts, such as by using compulsive behaviors. He was in fact stimulated by the homicidal thoughts, and the possibility of a paraphilic condition with sociopathy was also considered, but not all members of the psychiatric team were comfortable with making this determination.

It is also important to discuss the legal and ethical considerations of this case. While the patient was initially admitted voluntarily, the safety of the patient and others could be a strong indication for involuntary admission if the patient refused ongoing psychiatric assessment and treatment. The patient did agree to stay in the hospital voluntarily as he and his mother talked frequently about his need to get better in order to safely return home. Another consideration is the “duty to warn” and the involvement of law enforcement, which varies state to state. In the case of this patient, when the patient began to express explicit thoughts of harming a specific person, such as the unit staff member, that individual was informed and transferred to a role off the unit. Another ethical concern was the continuation of treatment in inpatient psychiatry due to improvements in his psychosis, with significant residual homicidal ideation. The implication is that the patient’s continued treatment could be done in an outpatient setting if psychosis was no longer present, as he might not have met inpatient criteria; however, this was not ethically possible due to safety concerns for the patient and potential named victims.

## Conclusions

Homicides have increased both locally and globally over the years, particularly among adolescents. This case report highlights the challenges of managing homicidal ideation in an 18-year-old male patient with comorbidities in an inpatient psychiatric facility. Legal and ethical considerations, such as decisions about involuntary admission and addressing the duty to warn, contributed to these challenges. Additionally, the patient’s history of traumatic experiences exacerbated the severity and persistence of his homicidal ideation. Pharmacological challenges and the need for personalized treatment strategies further complicated the treatment of his symptoms. Despite comprehensive treatment approaches, including psychopharmacology and electroconvulsive therapy (ECT), many of the patient’s symptoms persisted, illustrating the difficulty of addressing homicidal ideation. Further research is recommended for optimizing treatment in this emerging issue and related symptomology.

The limitations of this study include our insufficient knowledge of the patient’s full developmental history and the baseline of his social interactions. We suspect that additional family history may have been helpful but recognize that the patient’s mother was in a difficult role attempting to advocate for her son, as well as protect herself and her family. Additionally, we did not have access to pharmacogenetic testing or levels of aripiprazole that could help guide decisions regarding his drug metabolism profile and therefore medication dosing and choices.
